# Supplementary material for: Splice-Junction-Based Mapping of Alternative Isoforms in the Human Proteome
Source: Cell Rep. Author manuscript; Available in PMC 2020 Jan 15. (PMC6961840; doi:10.1016/j.celrep.2019.11.026)
Supplement: 2 [file NIHMS1546469-supplement-2.zip › DF1/PXD010154/Colon/Colon_8_MAP4_MYHDDDLADLVFPSSATADTSIFAGQNDPLKDSYVPLELAK.pdf]

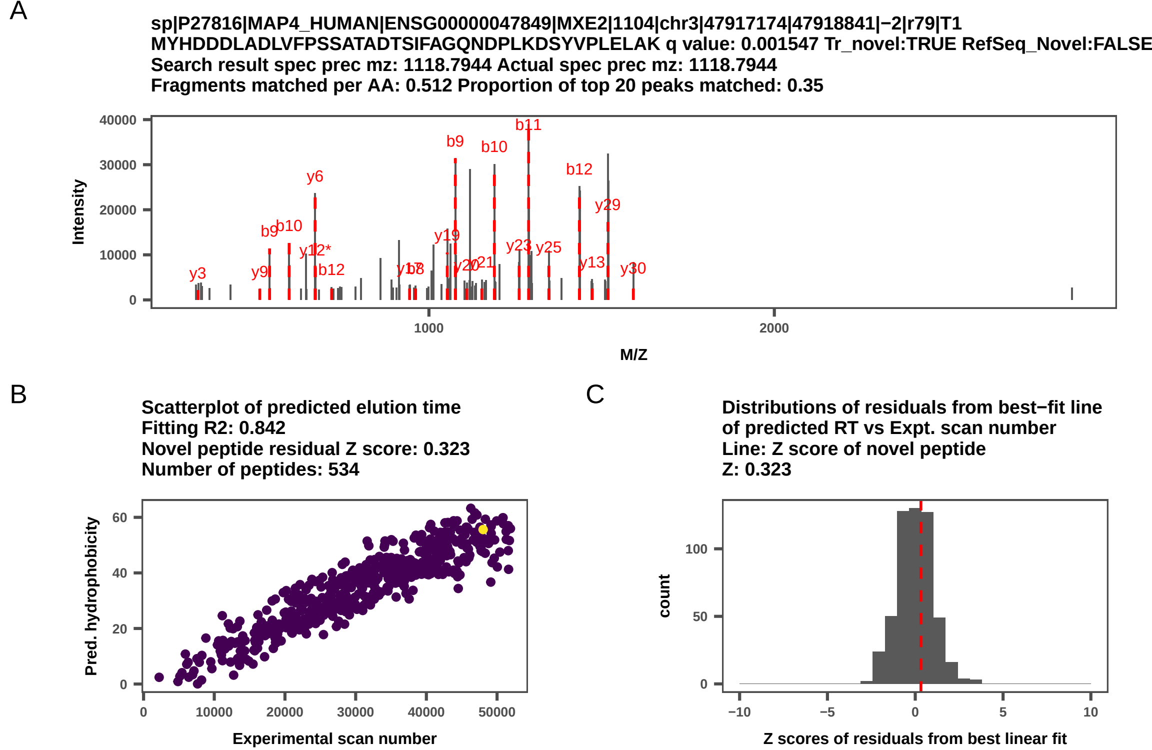
A
sp|P27816|MAP4_HUMAN|ENSG00000047849|MXE2|1104|chr3|47917174|47918841|−2|r79|T1
MYHDDDLADLVFPSSATADTSIFAGQNDPLKDSYVPLELAK q value: 0.001547 Tr_novel:TRUE RefSeq_Novel:FALSE
Search result spec prec mz: 1118.7944 Actual spec prec mz: 1118.7944
Fragments matched per AA: 0.512 Proportion of top 20 peaks matched: 0.35
Intensity
40000
30000
20000
10000
0
M/Z
1000
2000
y3
y9
b9
b10
y6
y12*
b12
y17
b8
b9
y19
y20
y21
b10
b11
y23
y25
b12
y13
y29
y30
B
Scatterplot of predicted elution time
Fitting R2: 0.842
Novel peptide residual Z score: 0.323
Number of peptides: 534
Pred. hydrophobicity
60
40
20
0
Experimental scan number
0
10000
20000
30000
40000
50000
C
Distributions of residuals from best−fit line
of predicted RT vs Expt. scan number
Line: Z score of novel peptide
Z: 0.323
count
100
50
0
Z scores of residuals from best linear fit
−10
−5
0
5
10
